# ‘It was Supposed to be a Secret’: a study of disclosure and stigma as experienced by adults with differences of sex development

**DOI:** 10.1080/21642850.2022.2102018

**Published:** 2022-07-22

**Authors:** Line Merete Mediå, Lena Fauske, Solrun Sigurdardottir, Kristin J. Billaud Feragen, Charlotte Heggeli, Anne Wæhre

**Affiliations:** a Women and Children’s Division, Centre for Rare Disorders, Oslo University Hospital, Oslo, Norway; b Department of Interdisciplinary Health Sciences, Institute of Health and Society, University of Oslo, Oslo, Norway; c Department of Oncology, Norwegian Radium Hospital, Oslo University Hospital, Oslo, Norway; d Educational Psychology Service, Tonsberg Municipality, Tonsberg, Norway; e Department of Child and Adolescent Psychiatry, Oslo University Hospital and Institute of Clinical Medicine, Oslo, Norway

**Keywords:** Conceal, disclosure, disorder of sex development, differences of sex development, intersex, stigma

## Abstract

**Background:**

Differences of sex development (DSD) are a group of congenital conditions that involve variations in sex chromosomes, genes, external and/or internal genitalia, hormones, and secondary sex characteristics. The present study sought to highlight the everyday challenges faced by adults with DSD as well as to understand how issues such as disclosure, information sharing, and stigma affect their daily life.

**Method:**

We applied an interpretative phenomenological study design to explore the first-person perspectives. Semi-structured qualitative interviews of 15 adults aged 30–70 years living in Norway with five different DSD conditions (Turner syndrome, Klinefelter syndrome, congenital adrenal hyperplasia, Mayer-Rokitansky-Küster-Hauser syndrome and hypospadias) were analyzed using reflexive thematic analysis.

**Results:**

Living with DSD, indicated doing a balancing act between hiding and/or exposing what participants perceived differed from others bodies. Communication regarding sensitive topics proved to be important. The participants were doing invisible work to manage the balance between concealing and revealing their feeling of differentness, a work effort that was not necessarily perceivable to others but still affected everyday life of the participants. Furthermore, the participants’ experiences of disclosure changed over time, as those who were diagnosed during childhood found that disclosure became easier with advancing age. However, being diagnosed as an adult seemed to increase the feeling of difference and complicate disclosure.

**Conclusion:**

Individuals with DSD should receive adequate information and have someone to practice disclosure towards, which could possibly strengthen the psychosocial aspects of living with their condition. The results emphasize the need to help individuals with DSD achieve a balance between disclosure and self-protection, overcome stigma, and determine when and how information about their DSD should be provided to others.

## Introduction

Differences of sex development (DSD), which are also referred to as disorders of sex development or intersex, are a group of conditions that involve variations in individuals’ sex characteristics, resulting in their genitals, hormones, or chromosomes differing from traditional conceptions of male and female bodies (Lee, Houk, Ahmed, & Hughes, 2006). The DSD population is large and heterogeneous with regard to diagnoses, severity of medical complications, psychological impacts, treatments, and follow-up (Kim & Kim, 2012; Lee et al., 2006). Some patients have visible variations in phenotype (e.g. in their genital appearance), while others exhibit differences in genotype (e.g. in their sex chromosomes). The estimated incidence of DSD varies from 1:200–1:300 (García-Acero, Moreno, Suárez, & Rojas, 2019), to 1:4500–1:5500 newborns (Sax, 2002). DSD are complex conditions that affect not only physiological processes within the body. Any chronic health condition can potentially affect the sense of identity and psychological well-being. In addition, treatments and the way others respond to bodily differences can have a negative impact on mental health in individuals with DSD. Knowledge and understanding of these impacts are important for those of us born with DSD.

In 2006, a consensus statement concerning the management of DSD was published, recommending that the evaluation and long-term care of people affected by DSD should be conducted at medical centers with multidisciplinary teams familiar with their medical needs (Lee et al., 2006). In Norway, multidisciplinary teams follow-up children with DSD until the age of 18. After the age of 18 years, there are no organized multidisciplinary follow-ups. Multidisciplinary DSD teams can provide psychological support to affected individuals and their families as a standard component of care, with children receiving age-appropriate medical information, and gender issues being discussed (Hiort et al., 2014).

The debate concerning the level of disclosure towards affected individuals, information sharing and multidiscipline follow-up of adults is not new within the field of DSD (Sutton et al., 2006; Tremblay, Van Vliet, Gonthier, & Janvier, 2016). Already in the 1950s, the debate about autonomy and shared decision-making, which is rooted in well-informed patients regarding their condition and bodily difference, was central as a consequence of the Nurnberg codex and the Geneva declaration (Reis, 2019), a debate that was ongoing for decades. In the 1980s, John Money (1987) wrote on the importance of educating patients about their condition in ways that might reduce the likelihood of it being received as stigmatizing. Stigma stems from undesirable attributes that people typically seek to avoid (Goffman, 1963, pp. 3–4).

In 2006, the consensus statement underscored the importance of full disclosure toward individuals with DSD (Howe, 2021; Lee et al., 2016). However, there still remained challenges in terms of sharing information about DSD, given the complexity of sexual development, the cultural impacts of DSD, and the sensitive nature of the information being shared (Lampalzer, Briken, & Schweizer, 2021; Malmqvist & Zeiler, 2010; McCauley, 2017; Weidler & Peterson, 2019). Moreover, advice concerning whether or not to share such information with a wider circle of people was recognized as a complicated issue (Hughes, Nihoul-Fekete, Thomas, & Cohen-Kettenis, 2007). In addition to challenges concerning disclosure, individuals with DSD may experience stigma in social contexts that may increase their communication difficulties (Earnshaw & Quinn, 2012). Recent studies have examined the effects of stigma on people with DSD and identified both feelings of shame (i.e. experienced stigma) (Engberg, Moller, Hagenfeldt, Nordenskjold, & Frisen, 2016) and withdrawal behavior (i.e. anticipated stigma) (Meyer-Bahlburg, Khuri, Reyes-Portillo, & New, 2017; Meyer-Bahlburg, Khuri, Reyes-Portillo, Ehrhardt, & New, 2018).

Qualitative research that gives voice to the individual experience and provides a more in-depth understanding of the everyday life of people with DSD is scarce (Lundberg, Donasen, Hegarty, & Roen, 2019; Roen, 2018). In particular, the 2006 consensus statement highlighted the need for research on adults’ experiences of living with DSD that could elucidate their everyday needs and improve the quality of healthcare over the course of life (Cools et al., 2018).

Given that DSD can negatively affect individuals’ psychosocial well-being and quality of life (e.g. due to a lack of information, difficulties with disclosure and stigma), there exists a pressing need to identify areas of life in which affected individuals might require additional support. In order to make greater use of valuable resources in the health care system, clinicians need to investigate adults` personal experiences of living with DSD. The overall aim of the present study was to describe the everyday challenges faced by adults with DSD and to explore how issues such as disclosure, information sharing, and stigma affect their daily life.

## Materials and methods

To achieve the aims of this study, we applied a qualitative and explorative design, and utilized a phenomenological and hermeneutical approach. Phenomenology is a systematic examination of different ways of experiencing reality. It seeks to explore and understand the lived experience of a phenomenon, and the way it is experienced and described by the individuals themselves (Kvale & Brinkmann, 2009). A hermeneutical approach implies a method used to understand and interpret the phenomena as expressed by participants (Kvale & Brinkmann, 2009). The approach is based on the participant’s and the researcher’s preunderstandings, on the context of the interviews, and develops throughout the entire research process (Gubrium, Holstein, Marvasti, & McKinney, 2012). We sought to understand how the participants described their experience of living with DSD, and how they perceived and spoke about their diagnosis to others by using semi-structured interviews as a tool.

We chose to include different diagnoses of DSD to shed lights on what was similar, or what distinguished the different diagnoses in adults aged 30–70 years.

### Participants

The total number of participants comprised a convenience sample of 15 adults. All participants expressed identifying with the sex they were assigned at birth (five males and ten females). They differed with regard to their diagnosis, age (range: 30–70 years, *M*_age_: 44 years), time of diagnosis made (children, adults), and number of surgeries. An approximate age range is reported for the purpose of de-identification. In the following, we present demographic descriptions for each diagnostic group represented in the material based on information from the participants in the semi-structured interview: Five of the female participants had Turner syndrome (TS). Of these, four were diagnosed within pre-pubertal age (range: 4–12 years) and one was diagnosed as adult. The group reported no surgical interventions related to DSD. Three female participants had congenital adrenal hyperplasia (CAH), and all were diagnosed within the first five years of life. In this group, all had undergone surgery on the genitalia within the first two years of life. Of these, two had undergone correctional surgery in pre-pubertal phase and/or as young adults. Two female participants had Mayer-Rokitansky-Küster-Hauser syndrome (MRKH) and were diagnosed in adolescence (range 14–18 years). Of these, one had undergone two operations, including a vaginoplasty, and the other had none. Three male participants had proximal hypospadias (referred to as hypospadias in text). They were all diagnosed within the first two years of life and had undergone surgery of genitalia within the first two years of life. Number of operations ranged from six to nine in these participants with hypospadias, including staged repairs and several reconstructions due to complications (range 6–40 years). Two male participants had Klinefelter syndrome (KS), one was diagnosed in early adulthood and the other in adulthood. Participants with KS reported no surgical interventions related to DSD.

All participants expressed identifying with the sex they were assigned at birth. The time towards the diagnosis of DSD was made, differed between and within diagnoses. Eleven participants were diagnosed before the age of 18, and four were diagnosed as adults from the age of 18 years to 35 years. See Table 1 for a brief description of the five diagnoses.

**Table 1.** Brief description of the represented conditions.

Diagnosis	Brief description	References
Congenital adrenal hyperplasia (CAH)	CAH affects both males and females. Persons born with CAH lack an enzyme that the body needs to produce cortisole and aldosterone, two vital hormones. Consequently, the body produces more testosterone than needed. For girls, this may result in genital variations such as a larger than typical clitoris and a closed vaginal opening. Persons diagnosed with CAH are in need of lifelong medication to normalize their hormone levels	Witchel (2017)
Mayer-Rokitansky-Küster-Hauser syndrome (MRKH)	MRKH affects only females. The ovaries and external genitals are normal and females with MRKH develop breasts and pubic hair. However, females born with MRKH have a uterus, cervix and upper vagina that has not developed as expected. Consequently, they do not start to menstruate and cannot become pregnant. Penetrating intercourse might be difficult because of a shorter vagina	Herlin, Petersen, and Brännström (2020)
Turner syndrome (TS)	TS only affects females. They lack partly or completely the one X chromosome. The most common future of TS is short stature and non-function ovaries, resulting in a lack of monthly periods and infertility. TS is often associated with a number of other health conditions and symptoms, including learning difficulties and social problems	Shankar and Backeljauw (2018)
Klinefelter syndrome (KS)	KS only affects males. Individuals with KF are born with an extra X-chromosome (XXY) and do not produce the usual level of testosterone. Males with KS have differences in the development of male characteristics (testes and body hair), delayed puberty and KS may affect bone strength and fertility	Tremblay et al. (2016)
Hypospadia,	Hypospadia only affects males and affects the development of the penis. The types of hypospadias range from the urethral opening appearing nearer the tip of the penis or nearer the scrotum The testis may be affected	Kumar and Cherian (2022)

All participants had received medical follow-ups in childhood. None reported receiving a multidisciplinary follow-up as adults. All of the participants spoke Norwegian as their native language. Individuals with a condition defined as DSD, as described by Lee et al. (2006), were invited to participate. The exclusion criterion was the presence of an intellectual disability that affected the ability to participate in the interview process (no individuals were excluded from the study).

### Procedure and measures

Clinicians representing the two multidisciplinary DSD teams in Norway contributed to identifying all eligible participants by searching for relevant diagnostic ICD-10 codes in their institutions’ electronic health records. In addition, two national competence centers for rare disorders contributed to identifying eligible participants from their registry. Patient support groups and four organizations for Lesbian, Gay, Bisexual, Transgender and Intersex (LGBTI) working for equal rights for people who challenge the norm for gender and sexuality, promoted to participation by spreading information about the study on their webpages and social media channels. All of the potential participants received written information about the study and a consent form by mail. After we received their signed consent forms, the participants were contacted by telephone to arrange a time and place for the interviews.

Each participant was offered the choice between a face-to-face interview and a telephone interview. Nine participants chose a face-to-face interview, while six participants opted for a telephone interview. The face-to-face interviews were conducted at the Oslo University Hospital in Norway, and the relevant participants’ travel expenses were reimbursed. All interviews were conducted from May to September 2018 by two female authors (A. W. and C. H.), a child psychiatrist who works in the field of DSD, and one with a master`s degree in psychology. The participants had never met the interviewers before. The interviews lasted between 45 and 90 min. The interviews were audio recorded using a Zoom H2n Handy Recorder and transcribed verbatim by CH and two research assistants. The participants were de-identified in the transcripts. The interview guide was designed to elicit accounts of the participants’ experience of everyday life with DSD. Participants were asked open-ended questions covering a wide range of themes, including romantic relationships, experienced discrimination, satisfaction with surgical / medical / psychological treatment, information received since diagnosis, and disclosure. The participants were encouraged to describe their experiences, and follow-up questions were used to prompt the participants to elaborate on relevant issues or to offer examples to illuminate their stories (Kvale & Brinkmann, 2009).

The study formed part of a larger research project commissioned by the Norwegian Directorate for Children, Youth and Family Affairs (Feragen, Heggeli, & Wæhre, 2019) which aimed to explore the group’s life situation and requirement for health and care services and interventions. The report is available in Norwegian with an English abstract and has not previously been published in English. A total of 334 invitations were distributed, and 83 signed consent forms were received (aged 18-70). The response was higher in some groups (TS and KS). A purposive sample of 27 people was drawn to have participants representing age, gender, geographical affiliation and diagnosis.

In the present study, we analyzed and reinterpreted data concerning a subset of the original adult sample involved in the main project. The 15 participants in our study were selected on the basis of their age (30 years and older). The reason for this was three-fold: first, we wanted to restrict the age range from the original 18–70 years; second, there is an ongoing parallel sub-study examining the lived experiences of young adults; and third, we wanted to dive deeper into the data than what had been done in the initial analysis that was more descriptive.

In order to secure patient and public involvement, a reference group was established as part of the larger research project that comprised user participants, LGBTI activists, patient organizations, and professionals with legal, medical, and psychological backgrounds. The reference group represented a variety of gender perspectives and medical and legal interests.

### Ethics

This study was conducted in accordance with the principles of the Declaration of Helsinki. All protocols and methods were approved by the Norwegian Regional Committee for Medical Research Ethics in South-Eastern Norway (number 79444) and by the Data Protection Officer at Oslo University Hospital (number 7000898). Due to the sensitive nature of the topics discussed during the interviews, the participants were offered a follow-up conversation after the interview. No one expressed a need for this.

### Data analysis

The qualitative data obtained from the interview transcripts were first assessed independently (L. M. M.) and then collaboratively by four of the authors (A. W., L. F., L. M. M., and S. S.). These authors comprised a group with a variety of professional background (a nurse, a clinical psychologist, a child psychiatrist and a researcher in medical humanities), of which two are working within the DSD field. The data analysis was drawn on both Braun and Clarke’s (2006) six-stage process and the principles of reflexive thematic analysis (Braun & Clarke, 2019). Reflexive thematic analysis is recognized as a suitable method for identifying patterns of meaning across datasets as well as divergence within data (e.g. between diagnosis, age at the time of diagnosis, and gender) (Braun & Clarke, 2019). Meaning requires interpretation, and it is not self-evident within data (Braun & Clarke, 2019). In this study, familiarization with the data was achieved through reading, re-reading, and making notes in the margins while striving to keep an open mind. The data were inductively coded (and recoded) by hand by the first author with the aim of identifying the participants’ personal and pre-reflexive experiences of disclosure (Braun & Clarke, 2006). First, the codes focused on each participant’s experiences as they appeared in the transcribed material. Next, the researchers searched for categories, similarities and divergences. In this process, we identified two themes. Drawing on these themes, we read through the interviews once more to notice how these two strategies were expressed by the participants. To elucidate each theme, the researchers provided a selection of illustrative quotations, which were slightly revised to improve the readability, in accordance with the approach of Kvale and Brinkmann (2009). Quotes from the interviews were translated from the original language into English. In what follows, pseudonyms are used to protect the confidentiality of the participants, and diagnosis and age range are reported to increase readability.

## Results

Participants represent a heterogeneous group of individuals affected by DSD. Two major themes were generated from the data regarding experiences of daily living: (a) Hiding a different body: A way of managing being different; and (b) Revealing information: From coerced exposing to acceptance of ambiguity.

### Hiding a different body: a way of managing being different

All participants disclosed a story about a body that functioned or had an appearance that in some way was different from most other bodies. Two sub-themes were generated: (a) Concealing a functionally different body; and (b) How am I perceived by others?

#### Concealing a functionally different body

Being born with the condition that affects sex development affects how the body works in different ways. Most people take body functions for granted, e.g. like standing and peeing for men, using a tampon, being pregnant or being able to carry out sexual intercourse with penetration. For the participants, functionality seemed to take a big focus of attention. At the same time, the effort involved in concealing their difference was not necessarily visible to other people, and sometimes not even to themselves, which suggests that this effort may be understood to comprise internalized actions they were unaware of. Such actions included, for example, detailed planning, making up excuses, and avoiding situations such as dating, sport activities, and using communal showers.

The male participants described the importance of having a penis that was functionally ‘normal’ and how this affected their everyday life. Peter described how he ‘took for granted’ (his words) what he did to compensate for not being able to stand up and pee without spilling urine, and how this is actually not something ‘normal’ people do:

This is why I like it when you can lock the door behind you at a public toilet, because then you can … (pauses), if you are peeing, you can clean up afterwards [ … ]. Because it is not cool if your buddies come in after you and see the mess [ …]. You think about this when you are out on the town, when you are at home and need to use the bathroom, when you are going to have sex, when you are going to work, taking a bath, everything (hypospadias, 30–49 years).

Two thirds of the participants spoke of taking precautions in relation to dating and intimacy, and how they struggled with such issues, dedicating a lot of mental resources to planning when it came to concealing their difference. Christian, one of the male participants with KS, discussed how having a small penis affected his relationships with women and how this resulted in avoidance behavior: ‘I can talk to women … that’s not the problem, but if it kind of advances to the next level, then I withdraw. It has to be really special before I take the next step’ (KS, 50–70 years).

Most participants seemed unaware of the invisible work they engaged in. Laura, one of the women born with CAH, stated: ‘In a way, there is nothing different about being born with this [CAH]. It’s the same as being born with a missing arm, or a heart condition, or something. Only, the consequences of it [CAH] can, of course, be very different’ (CAH, 30–49 years). When she talked about the consequences of CAH, she appeared to be referring to her virilized genitalia, which for many years caused her to believe that she was unable to have sex. For Lisa, the information she received about MRKH when being diagnosed (i.e. information about her vagina being too shallow and requiring vaginal dilatations), affected her sex life in a negative way. Before diagnosis she had an uncomplicated sex life, but afterwards it became difficult: ‘I was single for many years and dreaded having sex […] and it actually made me avoid sexual contact with men for several years’ (MRKH, 30–49 years).

#### How am I perceived by others?

Being born with a condition that affects sex development, not only affects the body’s functionality but may also affect what the body looks like and how others perceive you. In the same way, as participants avoided situations where someone could discover the different functionality with their body, they were afraid of being perceived as different. This fear seemed to affect the balance between concealing and revealing personal information.

Having thoughts about gender identity, and which gender role you possess or fit into both in the gaze of others and in your own view, is a part of living with DSD for some participants. Susanna discussed how lack of information from clinicians and parents and communication about physiological and psychological processes affecting CAH caused her to worry about who she was and where she fitted in: ‘You kind of felt like the identity-part was a bit difficult then. […] I am, well, what sex am I? In a way […]. Yes, it was probably during adolescence’ (CAH, 50–70 years). Although all of the participants expressed that they had reached a point in life where they were confident about their gender identity, they continued to feel afraid of how they might be perceived if their peers knew about the DSD condition. This made them avoid situations such as dating, sports, and using communal showers. Christian, mentioned how traveling with colleagues left him in a difficult position when it involved spending the night in a hotel with colleagues: ‘It was a nightmare if I had to share a room with one, two, or three others. We had to share showers and all that’ (Christian, KS, 50–70 years). He explained how he came up with excuses and told lies to avoid sharing rooms so that no one could reveal his bodily difference. The use of phrases such as ‘it was a nightmare’ indicates that sharing rooms may be understood as something that both threatened the concealment of his condition and created a lot of effort and lies.

The invisible work to avoid being revealed having a DSD condition affected the childhood and youth in a significant way and continued to affect the daily life as adults. Peter, who was a talented athlete, chose to discontinue as an athlete due to his fear of his atypical penis being revealed: ‘I avoided all sports, all team sports […]. I did not want to shower. No, it was a big deal. That was mainly the thing’ (hypospadias, 30–49 years). All of the male participants reported taking precautions when faced with having to use a communal shower as an adult, such as checking the locker room for separate showers or going home to shower after sports. The women on the other hand, seldom talked about public appearance, but more about how it affected intimate and private situations.

Most participants seemed unaware of the invisible work they engaged in, giving contradictory narratives. For instance, one male participant indicated that no partner had ever commented on the appearance of his penis and that he was content with the look of it. However, he expressed that he was unlikely to date girls in case they found out about his unusual genitalia.

### Revealing information: from coerced exposing to acceptance of ambiguity

The decision to reveal information about DSD consisted of dilemmas and a balancing act between a need to control what others might think of them and a need to tell about it. Three different subthemes were generated that influenced disclosure: (a) the context; (b) time of diagnosis; and (c) whether they mastered an everyday language.

#### To disclose, or not to disclose? Context matters

In this subtheme, participants reveal how they prefer to conceal personal information. Yet, in order to have intimate or close relations, they may see the need for disclosure or feel that it is necessary. Issues like fertility, an altered appearance of genitalia or difficulties with having sex were important issues in this regard and shared by most participants. Laura experienced a strained relationship to sex all her life. When she met a partner she became serious with, she felt obligated to reveal sensitive information about the parts of her body that were private and different:

Well, it was necessary when sex became an issue. I knew she would understand that something was a bit different [ … ]. I felt a strong need to explain everything, about the operation and all those things [ … ]. I didn’t want them to think … , or to get strange fantasies or anything. I rather they knew (CAH, 30–49 years).

Some of the participants expressed how disclosure was considered a positive thing, when the recipient of the disclosure had some knowledge and/or interest in the matter. Sebastian experienced that it was easier to talk to others living with illnesses. ‘The only one I can talk to is my father … because he also has a.. not a syndrome.. but a (diagnosis), so I can talk to him, [ … ] he understands’ (KS, 30–49 years).

For most participants born with KS, TS and MRKH, infertility was an issue. The balance between feeling responsible for informing their partner and feeling a need to conceal infertility was a dilemma. It influenced intimacy, romantic relationships and contact with friends and acquaintances. This could involve avoiding social events and situations where they expected questions regarding, e.g. pregnancy, but for a few, it meant an opportunity to speak about infertility hoping to normalize and reduce the stigma that surrounded not having children. Ella’s words describe the issue of infertility:

So I think it's kind of OK to tell about (infertility), because it’s a way to make sure that people don’t run after me and ask when I am going to have children all the time, because that's very tiring. [ … ] One of the few things I avoid in everyday life is baby-shower and stuff like that. It can get a little tough (MRKH, 40–50 years).

#### Disclosure as a time-dependent phenomenon

In spite of the heterogeneity of DSD, a dichotomy appeared through the participants’ experiences: those who learned about their condition during childhood or whether they had been diagnosed as adolescents or adults (during/after puberty).

Several participants who were diagnosed during childhood (*n *= 11) commented that as they grew older, they became more at ease with their body and diagnosis. This resulted in them reaching a level of acceptance of their differentness, which made revealing information about their bodily differences easier. As Thomas expressed: ‘Well, I think I would have had more issues with talking about it 15–20 years ago. I wasn’t as open as I am now’ (hypospadias, 30–49 years).

Four participants who were diagnosed with DSD later in life felt that disclosure became more difficult after being informed about their condition. Ingrid learned about the condition when she was an adult and had not been aware that she might be perceived as ‘different’. She started reading about TS: ‘And, I was shocked. [ … ] Abnormal breasts, and private parts, and … So I thought, thank god I was married, otherwise I wouldn’t had the guts to get involved with a man’ (TS, 50–70 years). Participants with KS described how the diagnosis generated an awareness of their bodily differences. It became problematic to reveal their bodies at the beach, at the gym, in dressing rooms, and in intimate situations.

I got a slap in the face then. So yes, I still struggle with it a bit, mentally, I actually do […]. When I was younger, I didn’t care, but now I have learned about [the consequences of KS], I struggle to take off the clothes on my upper body […]. I feel that sex might be a bit more difficult now. Also, because I’ve figured this out, I’ve realized that my testicles should be much larger (Sebastian, KS, 30–49 years).

#### Mastering an everyday language

Participants expressed how they did not have a way of talking about DSD so that others could understand. Some even expressed how they lacked knowledge about the condition and how this affected how they talked about it. Jane for instance, explains:

If they ask, I tell them that my body produces more testosterone than your body does, and that I need to take medications to balance this. Then they reply: «oh, ok». Because I … I cannot give them any more information, because I don’t know any more (laughs) (CAH, 30–49 years).

Some participants mentioned how their lives would have differed in a positive way if someone had taught them how to use an everyday language to communicate their DSD:

I didn’t tell anyone about it, didn’t communicate anything about hypospadias to anyone until I was 20, 19, maybe 18 years old. It was a big secret in a way. It just went like that. I think this is the reason why it’s important to encourage children to talk about it, because otherwise the problems will escalate, rather than you understanding that it is really not such a big deal (Peter, hypospadias, 30–49 years).

Participants may thus realize that gaining knowledge of DSD and having the ability to communicate about their condition might be primarily positive for their own understanding and well-being.

## Discussion

The main finding of this study concerned how adults with DSD struggled with reaching a balance between information sharing and concealment. The avoidance behaviors exhibited by several of the participants may imply that they anticipate stigmatization or that stigma was internalized. Stigma may be generated by what is defined as undesirable and discrediting attributes that people typically seek to avoid. Fear of stigmatization could possibly explain the ambivalent component of disclosure; several commented on disclosure as important, that children and youth should be trained in practicing disclosure, while they, in contrast, did not feel comfortable with revealing private information.

### Impact on everyday life

Challenges concerning the disclosure of information about their bodily difference are central to everyday life and constitute an important facet of the experience of living with DSD. Previous research has stated that children and adolescents living with DSD born before the consensus statement of 2006 have experienced too little information and inadequate communication about DSD (Howe, 2021; Lee et al., 2016). Based on this knowledge, it is not surprising that our participants struggled with disclosure. However, it is important to note that none of the participants had received a multidisciplinary follow-up as adults. They had all reached adulthood in 2006, an age group for whom no routine follow-up has been implemented so far. This may be one of the reasons why they continued to struggle in silence or used mental resources to decide whether to hide or reveal what they perceived as different.

A quantitative study including 1040 participants born with DSD found that a positive overall body image was associated with disclosure about one’s condition (van de Grift, Cohen-Kettenis, de Vries, & Kreukels, 2018). In our study, participants had an ambivalent relationship with disclosing information, even if disclosure usually was perceived as a positive experience. Disclosure concerning their different body was reported as unnecessary, except in situations where it would be visible. This ambivalence has also been described in other studies (Lampalzer et al., 2021; Sharratt, Williamson, Zucchelli, & Kiff, 2020) and may be related to feelings of stigma. Similar observations are documented in other populations affected by chronic and/or congenital conditions. The stigma surrounding HIV/AIDS is well known. Other conditions that affect intimate parts of the body, e.g. fecal incontinence, could also be comparable, as it represents a taboo with bodily functions that will only be discernable in certain situations (Chelvanayagam, 2014). Chelvanayagam (2014) describes how people with gastrointestinal conditions who are hyper-vigilant for signs of possible social rejection or discretization, may try to conceal the difference if possible, by using defensive or avoidance strategies. Perceived stigma related to the pressure to pass as able-bodied was also reported in a large qualitative analysis following an online survey including a whole range of different rare conditions (Munro, Cook, & Bogart, 2021) and has also been discussed in relation to visible conditions (Germain et al., 2021; Masnari et al., 2013). People with DSD and other chronic conditions with feelings of internalized stigma are less likely to discuss the taboo openly, may not possess the appropriate vocabulary, and may fear that healthcare providers treat them with prejudice and discrimination (Earnshaw & Quinn, 2012). Hence, feeling of stigma can result in a reluctance to access care.

The lack of understanding from friends, families and health personnel can increase feelings of loneliness and stigma. The limited knowledge among people in general about DSD, as well as their lack of knowledge about how genetic, gonadal, and hormonal factors can affect individuals with such conditions, may add to the burden faced by individuals with DSD. This should be taken under consideration when planning on how and what to tell others. In a qualitative study, Engberg et al. (2016) described how individuals with DSD considered their condition as too complex to explain to others. Research shows that this may result in communication difficulties with health professionals indicating the need to develop vocabulary that can be adapted to different situations (Sanders & Carter, 2015). Our findings indicate that patients, parents, and clinicians lacked an everyday language for talking about differences in bodies in general as well as the impacts of bodily differences on psychosocial aspects of daily life and body image in particular.

### Invisible work

The participants in the present study, who lacked the opportunity to talk about their diagnosis while growing up, later engaged in invisible work to achieve a balance between concealing and revealing their difference. Furthermore, this invisible work seemed to be an effort to remain in control of the information flow and to avoid being ‘revealed’ (i.e. anticipated stigma). In particular, experiences with an altered genital appearance or function caused difficulties in terms of sharing information about their bodily deviations because they did not have sufficient knowledge about their differences. This finding is in line with several other studies (Alderson, Madill, & Balen, 2004; Engberg et al., 2016; MacKenzie, Huntington, & Gilmour, 2009; Meyer-Bahlburg et al., 2017). For our female participants with TS, their invisible work seemed to be focused on short stature, social problems, and infertility, as shown within the TS literature (Nisbet, 2020; Sutton et al., 2006).

Participants’ accounts of doing preparations before revealing information about the condition is not unique to DSD. In a study done by Sharratt et al. (2020), participants with different visible but concealable conditions (e.g. skin conditions or burn scarring on parts of the body that may not necessary be visible), discussed different ways and situations in which they took control over the disclosure process, e.g. by selecting the timing, location, and level of disclosure. Preparing disclosure was a way of controlling what others knew about their condition and worked as a coping mechanism, as illustrated in the present study.

Conducting in-depth interviews appeared to be important in terms of reaching a better understanding of the complex relation between disclosure, stigma, and everyday life. The interview guide did not contain questions that explicitly focused on ‘stigma’. However, the participants frequently reported narratives of shame, differentness, and coping mechanisms, indicating that participants did not have an awareness of the invisible job they did.

### Time of diagnosis

A diagnosis received early or later in life revealed a discrepancy across two groups. As stated above, growing older had a positive impact on feelings of difference in participants diagnosed in childhood, leading to acceptance of their own identity and, in some situations, a reduction in invisible work. Interestingly, the greatest discrepancies between the diagnostic groups were for participants diagnosed as adults. The information they received about having DSD made talking to others, getting undressed in public, and participating in intimate relationships more difficult than prior to receiving a diagnosis. This was the case for males with KS and females with MRKH. Guntram (2013) investigated how women found out about their atypical sex development during adolescence and how they considered themselves as either ‘normally different’ or ‘differently normal’. This reflected how they understood the diagnosis; a source of stigmatization and medicalization, or a way to make sense of their new situation. A medicalized language may alienate the patient from the condition and make him/her feel that the body is somehow diseased and something that needs to be fixed, something others might have problems to accept. MacKenzie et al. (2009) suggested that people may develop acceptance of their differences when they learn about it, have someone to talk to, and receive support from family and friends. This suggests that the dissemination of information needs to be sensitive and customized, even when the patient is an adult. Our results also indicate that healthcare professionals should examine how new information is perceived and understood, and how it potentially influences the affected persons’ identity and psychological well-being.

### Strengths and limitations

Few studies have explicitly focused on how adults over the age of 30 experience living with DSD. The result derived from the present study could therefore be useful for clinicians and researchers to understand how it is being an adult with DSD, and how we can better help those who need it. The study has a qualitative approach, and a relatively heterogenic population. The diversity of diagnoses might limit the generalizability of the findings. However, the explorative and qualitative nature of the study made it possible to generate themes across the material but also deviations within the material and gave us rich and nuanced examples suitable to illuminate the experienced phenomena from the participants’ own perspectives. In this study, the interviews were conducted in 2018. To our knowledge, health care follow-up has not changed during the last four years or during the COVID pandemic. Therefore, we considered that current findings do not demonstrate any time-related impact on how participants may have experienced health care follow-ups differently compared to if the interviews were conducted today.

The participants in this study were all born before the 2006 consensus statement and since then there have been changes in psychological health care and follow-up. Thus, the present findings should not be generalized to younger age groups because children born with DSD after 2006 might have received a different multidisciplinary follow-up.

Despite involving LGBTI organizations in the recruitment, we did not receive any participants from these channels. In addition, during the recruitment phase, predominantly people with KS and TS expressed an interest in participating. To achieve balance in terms of diagnostic representation, a purposive sample was chosen, in which the spread with regard to age and geographical location was taken into account. Yet, the sample might have resulted in a selection bias as we had no background information about the severity of their condition or other potentially important factors when recruiting. The reference group was used to check for relevance, and they gave positive feedback on results being recognizable and relevant to their daily life. Finally, all of the participants were Caucasian. Future studies should aim to recruit individuals from different ethnic and racial backgrounds.

## Conclusion

It is important that people with DSD receive appropriate information and has someone to talk openly to about the psychosocial aspects of living with their condition. Individuals with DSD need lifelong multidisciplinary follow-up and renewed information that is adjusted to the timing of diagnosis, life situation, and psychological status. This requires sensitivity and pacing of information sharing from both families and clinicians, and the necessary information should be provided in an individually adapted and personalized manner.

Disclosure and communication about DSD during adulthood can create or enhance inherent feelings of distress, stigma and lack of belongingness. The participants’ anticipation of reactions to their differentness seemed to have a major impact on what they chose to share, even as adults. Silence may lead to both anticipated and internalized stigma and increase the suffering in individuals with DSD. By increasing awareness and reducing misconceptions in the community, clinicians can influence the feeling of being accepted. We also need to recognize the impact on everyday life of working to hide or choosing to disclose a body affected by DSD. Awareness about differences such as DSD need to be dealt with at an interpersonal level, a community level, and last, but not least, on an institutional level so that people with DSD may avoid being exposed to attitudes representing outdated knowledge towards individuals with diversity in sex development.

## Data Availability

Due to the qualitative nature of this research, the datasets generated during the current study are not publicly available due to participant confidentiality issues.
